# *In Vivo* Emergence of a Novel Protease Inhibitor Resistance Signature in HIV-1 Matrix

**DOI:** 10.1128/mBio.02036-20

**Published:** 2020-11-03

**Authors:** Rawlings Datir, Steven Kemp, Kate El Bouzidi, Petra Mlchocova, Richard Goldstein, Judy Breuer, Greg J. Towers, Clare Jolly, Miguel E. Quiñones-Mateu, Patrick S. Dakum, Nicaise Ndembi, Ravindra K. Gupta

**Affiliations:** a University College London, London, United Kingdom; b Department of Medicine, University of Cambridge, Cambridge, United Kingdom; c Department of Microbiology and Immunology, University of Otago, Dunedin, New Zealand; d Institute for Human Virology, Abuja, Nigeria; e Institute of Human Virology, University of Maryland School of Medicine, Baltimore, Maryland, USA; f Africa Health Research Institute, Durban, South Africa; Medical School, University of Athens

**Keywords:** HIV, resistance, protease, drug, Africa, antiretroviral, Gag, antiretroviral resistance, human immunodeficiency virus, protease inhibitors, proteases

## Abstract

Protease inhibitors (PIs) are the second- and last-line therapy for the majority of HIV-infected patients worldwide. Only around 20% of individuals who fail PI regimens develop major resistance mutations in protease. We sought to explore the role of mutations in *gag*-*pro* genotypic and phenotypic changes in viruses from six Nigerian patients who failed PI-based regimens without known drug resistance-associated protease mutations in order to identify novel determinants of PI resistance. Target enrichment and next-generation sequencing (NGS) with the Illumina MiSeq system were followed by haplotype reconstruction. Full-length Gag-protease gene regions were amplified from baseline (pre-PI) and virologic failure (VF) samples, sequenced, and used to construct *gag*-*pro*-pseudotyped viruses. Phylogenetic analysis was performed using maximum-likelihood methods. Susceptibility to lopinavir (LPV) and darunavir (DRV) was measured using a single-cycle replication assay. Western blotting was used to analyze Gag cleavage. In one of six participants (subtype CRF02_AG), we found 4-fold-lower LPV susceptibility in viral clones during failure of second-line treatment. A combination of four mutations (S126del, H127del, T122A, and G123E) in the p17 matrix of baseline virus generated a similar 4-fold decrease in susceptibility to LPV but not darunavir. These four amino acid changes were also able to confer LPV resistance to a subtype B Gag-protease backbone. Western blotting demonstrated significant Gag cleavage differences between sensitive and resistant isolates in the presence of drug. Resistant viruses had around 2-fold-lower infectivity than sensitive clones in the absence of drug. NGS combined with haplotype reconstruction revealed that resistant, less fit clones emerged from a minority population at baseline and thereafter persisted alongside sensitive fitter viruses. We used a multipronged genotypic and phenotypic approach to document emergence and temporal dynamics of a novel protease inhibitor resistance signature in HIV-1 matrix, revealing the interplay between Gag-associated resistance and fitness.

## Abstract (precis)

Protease inhibitors (PIs) are the second- and last-line therapy for the majority of HIV-infected patients worldwide. Only around 20% of individuals who fail PI regimens develop major resistance mutations in protease. We sought to explore the role of mutations in *gag*-*pro* genotypic and phenotypic changes in viruses from six Nigerian patients who failed PI-based regimens without known drug resistance-associated protease mutations in order to identify novel determinants of PI resistance.

## INTRODUCTION

As global scale-up of antiretroviral therapy (ART) progresses in the absence of universal viral load monitoring, significant numbers of persons living with HIV (PLWH) are experiencing virological failure (VF) with emergent drug resistance (1–3). In addition, pretreatment drug resistance (PDR) has been rising over the past decade (4–6). Although integrase inhibitors are now recommended by WHO in regions where PDR exceeds 10% (7, 8), second-line ART in low- and middle-income countries (LMIC) is likely to remain dependent on boosted protease inhibitors (PI), specifically lopinavir/ritonavir or atazanavir/ritonavir.

Studies demonstrate that the detection of major canonical protease mutations (9) is around 20% in PLWH treated with PI-containing combination ART (10, 11), raising the question of how virologic failure occurs in the remaining cases. Inadequate adherence to medication has been implicated (12–14), and the contribution of minor protease mutations has been explored (15). Determinants of susceptibility outside the protease gene have also been considered (16). Interestingly, although PI monotherapy can be effective in some populations in clinical practice (17), this is associated with a higher prevalence of major PI resistance mutations at VF than PI combined with 2 two nucleoside reverse transcriptase inhibitors (NRTI) (18, 19).

The HIV-1 envelope (Env) has been reported in two studies to impact PI susceptibility (20, 21), with a number of reports of diverse *env* sequence changes during PI failure (22, 23). Gag is highly polymorphic across HIV-1 subtypes, and existing literature reports diverse mutations occurring both within and outside cleavage sites following treatment with older PIs, such as indinavir, saquinavir, and nelfinavir, in subtype B infections (16, 22–26). Although there is very limited information on the role of HIV-1 *gag* in susceptibility to modern boosted protease inhibitors, such as lopinavir/ritonavir, used in second-line ART for non-B subtypes, we and others have reported that around 1 in 6 individuals infected with non-subtype B HIV who fail modern PI have *gag*-encoded reduced phenotypic susceptibility to PI (27–31), though specific amino acid determinants have remained elusive.

Cleavage site mutations are thought to partially restore efficient cleavage by protease in the presence of bound drug (32, 33). The mechanism for non-cleavage site mutations may include allosteric changes in protease-Gag interactions that influence the efficiency by which protease locates cleavage sites through dynamic intermolecular interactions in the presence of drug (34, 35). For example, our group previously reported the emergence of T81A in Gag that appeared to correlate with reduced susceptibility to the modern PI lopinavir in a subtype AG-infected individual in France (28). This mutation was predicted to impact intermolecular interactions between Gag and protease by Deshmukh and colleagues using nuclear magnetic resonance (NMR) (35).

Here, we sought to explore the role of mutations in *gag*-encoded determinants of reduced PI susceptibility in non-subtype B HIV-1 and to elucidate their evolution in PLWH in Nigeria.

## RESULTS

### Phenotypic drug susceptibility following PI failure.

Participant characteristics of the six HIV-infected individuals failing PI-based second-line ART are shown in Table 1. Three were infected with CRF02_AG recombinant strain and three with subtype G HIV strains. Next-generation sequencing (NGS) of *gag* and *pol* was used to generate consensus sequences for the six patients at two time points: before PI treatment (baseline) and at virologic failure (VF). A significant number of amino acid changes occurred between time points in each individual, with most occurring in the matrix (p17) domain of Gag. Phenotypic PI susceptibility testing was performed on plasma-derived clones obtained at the same time points.

**TABLE 1:** Participant and virus characteristics

Patient (subtype)	Time point	Viral load (copies/ml)	Time between baseline and VF sample (mo)
Patient 1 (CRF02_AG)	Baseline	140,991	34
	VF	6,193	
Patient 2 (G)	Baseline	20,178	42
	VF	117,942	
Patient 3 (CRF02_AG)	Baseline	271,974	50
	VF	74,224	
Patient 4 (CRF02_AG)	Baseline	24,693	36
	VF	32,683	
Patient 5 (G)	Baseline	274,504	31
	VF	16,304	
Patient 6 (CRF02_AG)	Baseline	18,056	64
	VF	66,277	

**FIG 1:**
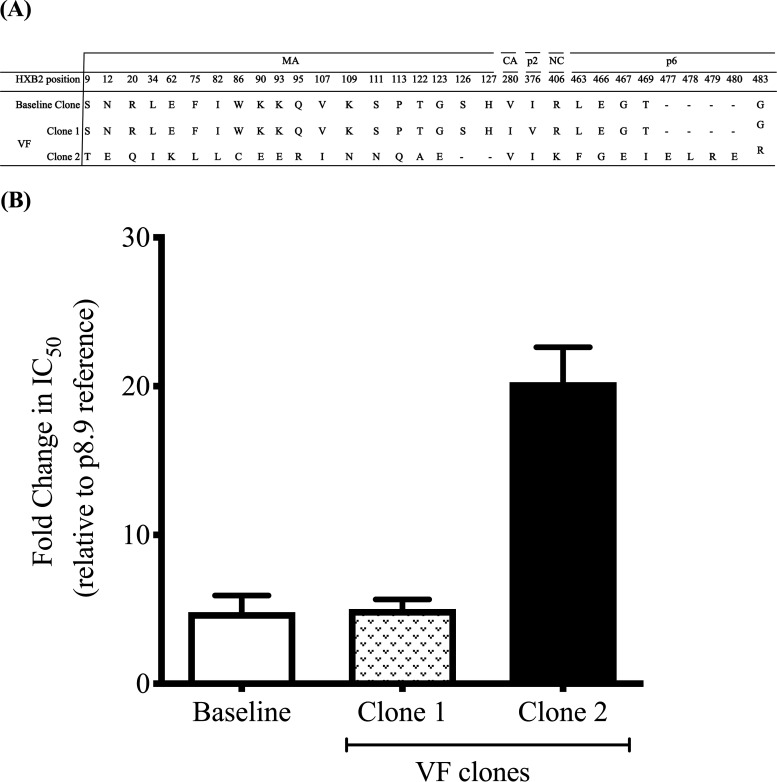
Variation in phenotypic PI susceptibility of full-length Gag-protease from HIV-1 infected patient at different time points. (A) Sequences of the viral clones showing the amino acid changes in the MA, CA, P2, NC, p1, and p6 regions of Gag between baseline (pre-PI treatment) and viral failure (during PI treatment). (B) Full-length Gag-protease sequence was amplified from plasma samples and cloned into p8.9NSX+. VSV-G pseudotyped viruses encoding luciferase were produced by cotransfection in 293T cells. The PI susceptibility of pseudovirions derived from each patient was measured by luciferase activity, as determined using a single-replication-cycle drug susceptibility assay. Data are fold differences in IC_50_s of LPV in comparison to that for the assay reference strain, p8.9NSX. Error bars represent standard errors of the means from at least three independent experiments performed in duplicate.

Participant 6 had a significant difference in PI susceptibility between baseline and failure time points (Fig. 1 and Fig. S1). At VF, the difference in the 50% inhibitory concentration (IC_50_) for lopinavir (LPV), expressed as fold change (FC) compared to the subtype B reference, was 20.3 compared to 5.2 prior to initiation of LPV treatment. We phenotyped four clones from baseline, all with similar LPV susceptibility. Baseline genotype (pre-PI) indicated that the individual had developed extensive resistance to first-line ART, with the nucleoside reverse transcriptase inhibitor (NRTI) mutations K65R and M184I conferring high-level tenofovir and lamivudine resistance, respectively, as well as K103N and Y181C conferring resistance to nonnucleoside reverse transcriptase inhibitors (NNRTI). The co-occurrence of the latter two NNRTI mutations suggests that the individual may have been pretreated with first-line ART containing nevirapine or have received single-dose nevirapine for prevention of mother-to-child transmission (36).

10.1128/mBio.02036-20.1 FIG S1 Phenotypic drug susceptibility to lopinavir of virus isolates at baseline (before second-line treatment) and after failure of second-line PI-based ART. Susceptibility is expressed as fold change in IC_50_ compared to a subtype B reference in a single-round assay. Error bars represent standard errors of the means from at least two independent experiments performed in duplicate. VF, viral failure. Download FIG S1, DOCX file, 0.1 MB. Copyright © 2020 Datir et al. 2020 Datir et al. This content is distributed under the terms of the Creative Commons Attribution 4.0 International license.

We further explored virus from this participant in order to elucidate determinants of resistance. Sequence alignment of full-length *gag* and protease genes from sensitive and resistant clones revealed 19 amino acid changes in matrix (MA), one change each in capsid (CA), p2, and nucleocapsid (NC), and an insertion of four amino acids (E, L, R, and E) at *gag* position 477 in the p6 region of the resistant clone (Fig. 1). In protease, there was an M46V mutation in the resistant virus that was found to have no impact on LPV susceptibility (Fig. S2).

10.1128/mBio.02036-20.2 FIG S2 Impact of protease mutation M46V on LPV susceptibility. Mutations were made in both sensitive and resistant viruses. Data are fold differences in IC_50_s of LPV in comparison to that for the assay reference strain, p8.9NSX. Error bars represent standard errors of the means from at least two independent experiments performed in duplicate. Download FIG S2, DOCX file, 0.1 MB. Copyright © 2020 Datir et al. 2020 Datir et al. This content is distributed under the terms of the Creative Commons Attribution 4.0 International license.

Interestingly, the VF sample was taken 64 months after PI initiation, when the viral load was 66,277 copies/ml, and within this plasma sample two distinct virus clones were isolated (Fig. 1B, hatched and black bars). There was a 4- to 5-fold difference in LPV susceptibility between the two clones, suggesting a mixture of susceptible and “resistant” viruses at the failure time point (Fig. 1). We proceeded to map determinants of susceptibility using these two clones identified at failure. First, we sought to determine the role of a four-amino-acid insertion in the p6 domain. Using standard site-directed mutagenesis techniques, amino acids E, L, R, and E were inserted into a susceptible clone at position 477 in the p6 domain (Fig. S3). Conversely, E, L, R, and E residues were deleted in the less susceptible clone from the same location. There was no significant change in susceptibility to LPV as a result of the ELRE insertion (Fig. S3).

10.1128/mBio.02036-20.3 FIG S3 Role of the four-amino-acid sequence in p6 on LPV susceptibility. (A) Amino acid sequence alignment showing positions in a *gag-pro* sequence that were mutated by site-directed mutagenesis (in red). (B) Fold change in LPV IC_50_ in a single-round assay. Error bars represent standard errors of the means from at least two independent experiments. Download FIG S3, DOCX file, 0.1 MB. Copyright © 2020 Datir et al. 2020 Datir et al. This content is distributed under the terms of the Creative Commons Attribution 4.0 International license.

### Matrix deletion of S126 and H127 confers reductions in LPV susceptibility.

**FIG 2:**
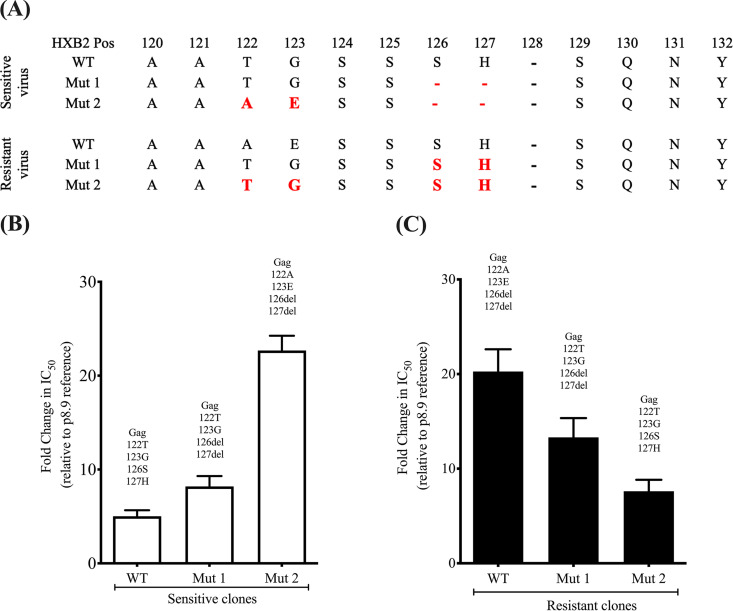
Gag 126del and 127del mutations occurring with T122A and G123E confer resistance to the protease inhibitor lopinavir in the absence of any major protease mutations. (A) Sequences of the viral clones showing the amino acid changes (in red) introduced using standard site-directed mutagenesis techniques. (B and C) Full-length Gag-protease with the indicated mutations was amplified from plasma samples and cloned into p8.9NSX+. VSV-G-pseudotyped viruses encoding luciferase were produced by cotransfection in 293T cells. PI susceptibility of pseudovirions derived from each patient was measured by luciferase activity, as determined using a single-replication-cycle drug susceptibility assay. Data are fold differences in IC_50_s of LPV in comparison to that for the assay reference strain, p8.9NSX. Error bars represent standard errors of the means from at least three independent experiments performed in duplicate.

Given that the greatest number of changes occurred in the MA region, we sought to explore a possible role for MA amino acid changes in PI susceptibility. First, sequence changes occurring near the MA/CA cleavage site (within 10 amino acids) were considered. We noted that the more resistant virus had a deletion of Gag positions 126 and 127 as well as adjacent T122A and G123E mutations. Using site-directed mutagenesis, serine (Gag position 126) and histidine (Gag position 127) residues were deleted in the susceptible clone. Conversely, serine and histidine residues were inserted in the less susceptible clone. Deletion of Ser-126 and His-127 in the susceptible virus led to a significant decrease in LPV susceptibility for the mutant virus (Fig. 2). Conversely, the insertion of Ser and His residues in the resistant virus increased susceptibility of the mutant (Fig. 2). However, the changes at positions 126 and 127 did not completely account for the differences in LPV susceptibility.

### The matrix deletions of S126 and H127 act synergistically with T122A and G123E in Gag.

A combination of S126del, H127del, and the T122A and G123E mutations in the susceptible virus led to a 4-fold decrease in susceptibility to LPV (FC in IC_50_ from 5.3 to 22.7) (Fig. 2 and Table S2). Conversely, S126ins, H127ins, and the A122T and E123G substitutions in the LPV-resistant virus led to a 3-fold decrease in resistance (Fig. 2). We also tested the effect of the four-amino-acid signature on susceptibility to the second-generation PI darunavir (DRV) and found no significant impact (Fig. S4).

10.1128/mBio.02036-20.4 FIG S4 Gag 126del and 127del mutations occurring with T122A and G123E did not have a significant resistance effect on susceptibility to the protease inhibitor darunavir. (A) Sequential introduction of mutations in the more susceptible clone (VF2 clone 1). (B) Reversion of the mutations in the resistant clone (VF2 clone 2). The sequences of the viral clones showing the amino acid changes introduced using standard site-directed mutagenesis are shown in red. Full-length Gag-protease amplified from plasma samples as well as respective mutants was VSV-G pseudotyped, and viruses encoding luciferase were produced by cotransfection in 293T cells. PI susceptibility of pseudovirions was measured by luciferase activity, as determined using a single-replication-cycle drug susceptibility assay. Data are fold differences in IC_50_s of LPV in comparison to that for the assay reference strain, p8.9NSX. Error bars represent standard errors of the means from at least two independent experiments performed in duplicate. Download FIG S4, DOCX file, 0.4 MB. Copyright © 2020 Datir et al. 2020 Datir et al. This content is distributed under the terms of the Creative Commons Attribution 4.0 International license.

10.1128/mBio.02036-20.6 TABLE S1 Genotypic consensus-level sequence data derived from next-generation sequencing for six individual participants failing protease inhibitor-based second-line ART before (BL) and after virological failure (VF). Amino acids indicated represent differences from subtype B reference strain NL4.3. Amino acid positions in bold represent those previously associated with exposure to protease inhibitors. Download Table S1, DOCX file, 0.3 MB. Copyright © 2020 Datir et al. 2020 Datir et al. This content is distributed under the terms of the Creative Commons Attribution 4.0 International license.

10.1128/mBio.02036-20.7 TABLE S2 Representative raw IC_50_ data for sensitive (WT) and mutant (Mut2 with T122A, G123E, S125del, and H126del) clones. Two replicates (1 and 2) are indicated with means and standard deviations. Download Table S2, DOCX file, 0.01 MB. Copyright © 2020 Datir et al. 2020 Datir et al. This content is distributed under the terms of the Creative Commons Attribution 4.0 International license.

We sought to establish the effect of each of the four amino acid changes occurring alone. Using the resistant viral clone, four different mutant viruses were created with single amino acid changes in Gag: A122T, E123G, S126ins, and H127ins. Results of the phenotypic drug susceptibility testing of these mutants showed that only E123G appeared to increase susceptibility (Fig. S5), and the combination of the four amino acids had the greatest impact on LPV susceptibility.

10.1128/mBio.02036-20.5 FIG S5 Role of single amino acid changes on LPV susceptibility. (A) Amino acid sequence alignment showing positions in a *gag-pro* sequence that confer “resistance” mutated by site-directed mutagenesis (in red). (B) Fold change in LPV IC_50_ in a single-round assay using luciferase as a readout. Error bars represent standard errors of the means from at least two independent experiments. Download FIG S5, DOCX file, 0.3 MB. Copyright © 2020 Datir et al. 2020 Datir et al. This content is distributed under the terms of the Creative Commons Attribution 4.0 International license.

**FIG 3:**
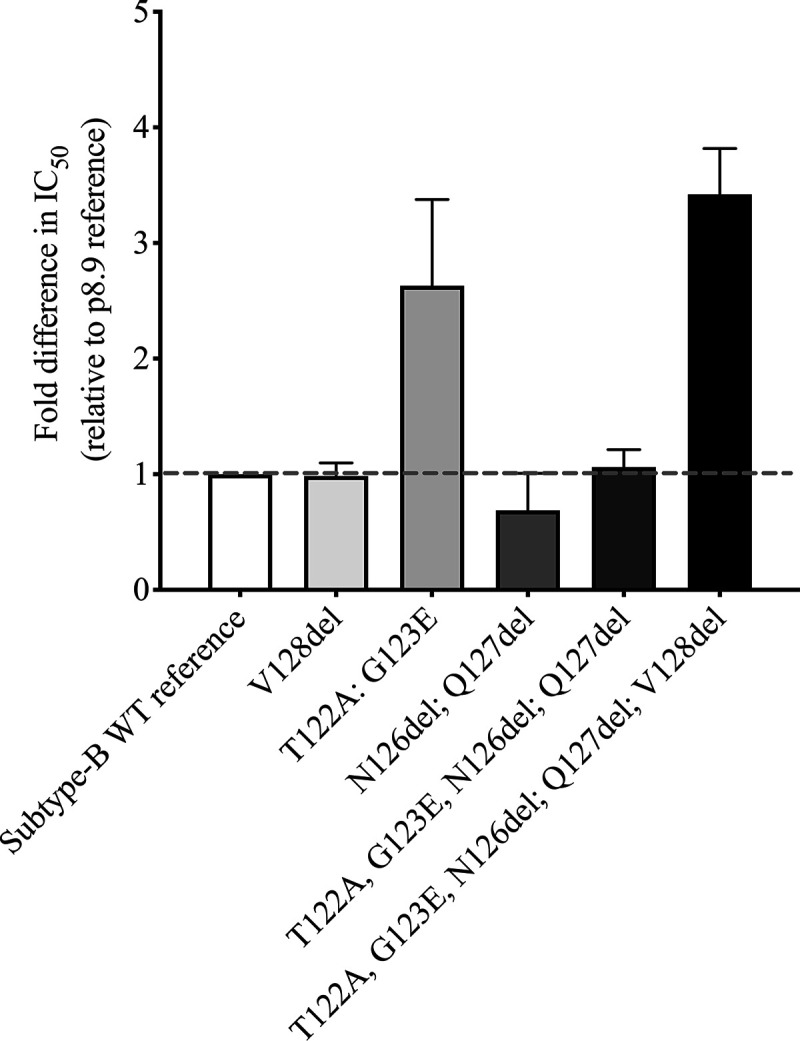
The four-amino-acid MA mutant signature can be introduced into subtype B to reduce PI susceptibility. Site-directed mutations were generated in the subtype B reference strain used in our assays. V128del was also added, as this deletion is present in HIV-1 CRF02_AG. Data are fold differences in IC_50_s of LPV in comparison to that for the assay reference strain, p8.9NSX. Error bars represent standard errors of the means from at least two independent experiments performed in duplicate.

We next tested whether the four-amino-acid signature T122A/G123E/S126del/H127del could confer LPV resistance in a different subtype context. We chose the reference p8.9NSX subtype B virus and made the amino acid deletions at Gag positions 126 and 127 as well as the adjacent T122A and G123E mutations. In addition, we added a V128 deletion, given that the subtype CRF02_AG consensus contains this deletion compared to subtype B. The five mutations (T122A/G123E/S126del/H127del/V128del) reduced susceptibility to LPV more than 3-fold, indicating that they are effective in a divergent subtype (Fig. 3).

### Matrix/capsid (p17-p24) cleavage and differential PI susceptibility.

**FIG 4:**
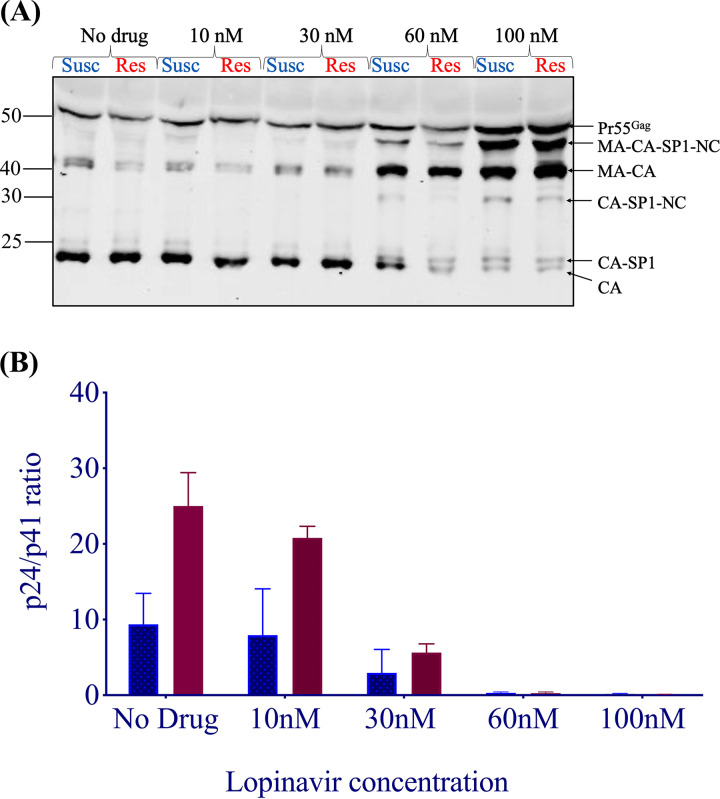
HIV-1 Gag cleavage efficiency in resistant (Res) versus susceptible (Susc) isolates. (A) Representative Western blot of virus-containing supernatant at increasing drug doses, using a p24 antibody. Mass (in kilodaltons) is indicated on the left. MA, matrix (p17); CA, capsid (p24); NC, nucleocapsid; SP1, spacer peptide 1. (B) Ratios of p24/p41 at increasing drug doses. Data are means and standard deviations from 2 independent experiments. In each pair, the left bar represents the wild type and the right bar represents resistant virus.

We hypothesized that the efficiency of MA/CA cleavage of HIV-1 polyproteins would differ between the susceptible and resistant clones in the presence of LPV. To test this hypothesis, we employed Western blot analysis. Gag cleavage patterns were examined using the supernatants and cellular extracts of 293T cells transfected with each plasmid in the presence and absence of increasing concentrations of LPV (Fig. 4). We probed with a polyclonal p24 antibody, and as expected, there was incomplete cleavage of p24-p2 at higher LPV doses in the virus-containing supernatants and the cell extracts, consistent with previous data (32). We calculated p24/p41 ratios to specifically probe the p17/p24 cleavage site in the vicinity of the four-amino-acid signature. We found that the resistant virus cleaved p17/p24 more efficiently in the absence of drug and up to 30 nM LPV.

### The resistance signature arises from a minority viral population detected at baseline.

We proceeded to investigate when resistance emerged. Given the lengthy period of over 5 years between the two samples, we ideally needed a sample from an intermediate time point. We were able to identify a plasma sample from a patient on second-line therapy from 41 months, with a VL of 241,894 copies/ml. We refer to the 41-month time point as VF1 and the original 64-month time point as VF2. NGS analysis at the whole-genome level was undertaken for all 3 time points, and Table 2 shows variant frequencies at sites in Gag and Pol associated with drug exposure. Of note, we observed loss of mutations affecting susceptibility to lamivudine (M184I), tenofovir (K65R), and efavirenz (K103N) between baseline and VF1. The individual was prescribed lamivudine, zidovudine, and lopinavir/ritonavir for second-line therapy, and the resistance data indicate lack of drug pressure from lamivudine.

**TABLE 2:** NGS variant-derived data for three time points during LPV treatment

Gene	Mutation^*a*^	% of reads encoding mutation at^*b*^:		
		Baseline (0 mo) (405,158)	VF1 (41 mo) (250,932)	VF2 (64 mo) (604,157)
Gag	E12K	5	21	42
	R76K	0	0	2
	Y79F	0	0	2
	T122A	4.8	13	26
	G123E	5.0	13	26
	V128del	100	100	100
	V370A	5	0	1
	S373T	97	98	100
	R409K	3	0	1
	S451T	100	1.7	0

RT	K65R	98	0	0
	K103N	94	0	0
	E138K	0	0	4
	Y181C	100	0	0
	M184I	100	1.2	21
	M184V	0	0	79

a Gag mutations known to be associated with protease inhibitor exposure from prior reports and resistance-associated mutations in RT.

b Numbers in parentheses are total numbers of reads.

The NGS showed that T122A and G123E were present at low abundance before initiation of PI (approximately 5% of reads) (Table 2). The proportion of T122A/G123E increased at VF1 to 13%. These mutations were observed at increased frequency at VF2 both by target-enriched NGS and also direct *gag*-*pro* PCR from plasma, but NGS also showed emergence of the lamivudine resistance mutation M184V, suggesting improved adherence to the lamivudine regimen between VF1 and VF2.

**FIG 5:**
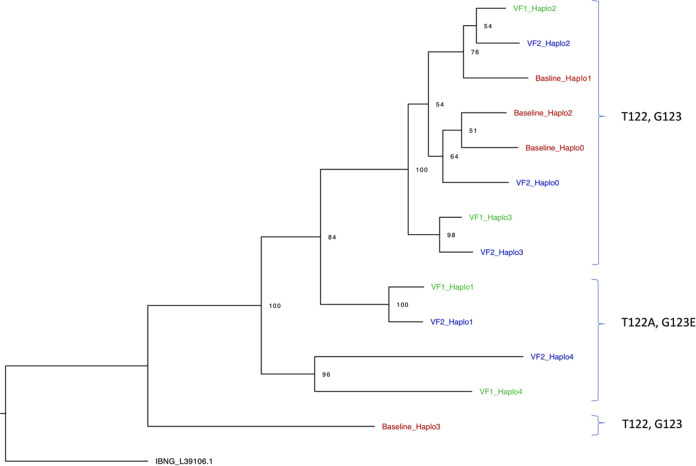
Whole-genome HIV haplotype reconstruction using target-enriched NGS Illumina MiSeq data from each time point (baseline, VF1, and VF2), with maximum-likelihood analysis and bootstrap support indicated using 1,000 replicates. Labels on the right are the amino acids at Gag positions 122 and 123.

We next generated whole-genome haplotypes for each time point using NGS data in order to first establish the phylogenetic relationships between viruses with differing PI resistance-associated mutations, and also to determine the coreceptor usage of virus haplotypes, as this might provide clues to the origins of virus variants (Fig. 5). All inferred haplotypes were predicted to use CCR5 with a false-positive rate (FPR) of <5%, and no CXCR4-using viruses were predicted in either of the two algorithms used.

**FIG 6:**
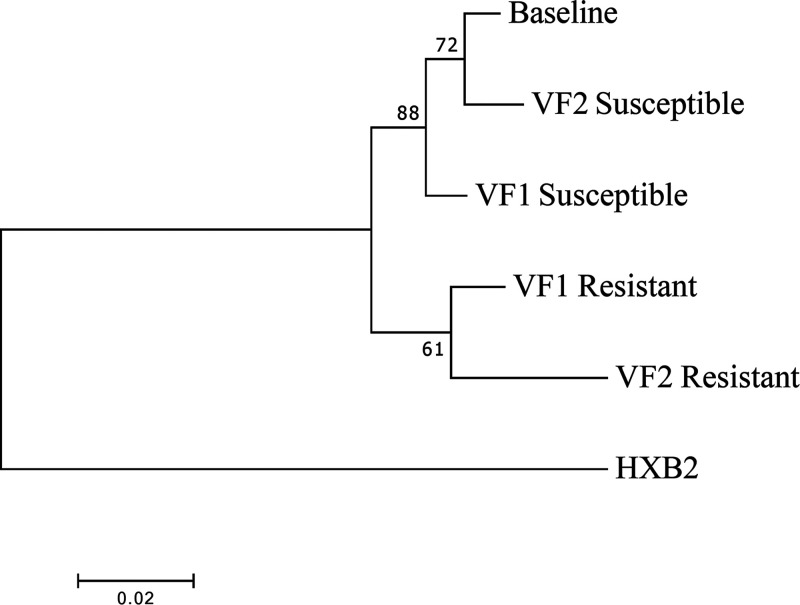
Phylogenetic relationships between viral Gag-protease plasma-derived sequences isolated at baseline (pre-PI) and at two failure time points (VF1 and VF2). The maximum-likelihood tree has bootstrap support indicated at the nodes. The outlier is HXB2, a subtype B virus. VF1, viral failure 1 at 41 months after initiation of protease inhibitor therapy; VF1, viral failure 2 at 64 months after initiation of protease inhibitor therapy.

We proceeded to clone sequences from plasma at VF1 in addition to those previously cloned from VF2 and inferred phylogenetic trees. None of the four *gag*-*pro* clones from baseline (before initiation of PI) contained any of the four amino acid changes (T122A, G123E, S126del, and H127del), consistent with NGS data showing that these variants were present at <5% (Table 2). Clones from the intermediate time point VF1 clustered with the VF2 clones rather than with the baseline clones (Fig. 6). Overall, there was excellent concordance between the inferred whole-genome haplotypes and *gag*-*pro* clones, though there appeared to be greater diversity in haplotypes. *In vitro* phenotypic drug susceptibility of cloned sequences revealed both sensitive and resistant viruses at VF1 as well as VF2 (Fig. 6), with the resistant clones from VF1 and VF2 clustering together and sharing the 4-amino-acid resistance-associated signature S126del/H127del/T122A/G123E. As expected, the susceptible clones from VF1 and VF2 also clustered with each other in a distinct part of the tree.

### Persistence of both resistant and susceptible viruses can be explained by replication capacity.

**FIG 7:**
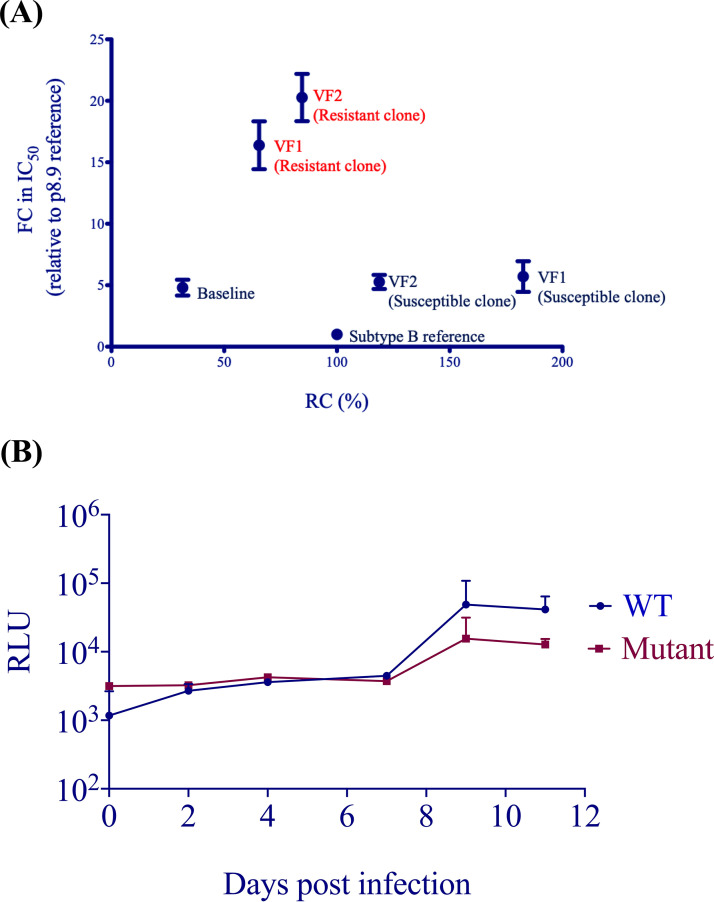
(A) Relationship between single-round infectivity (RC) and LPV susceptibility (FC in IC_50_ compared to subtype B reference) for single-round VSV-G-pseudotyped viruses bearing patient-derived Gag-protease gene sequences. Error bars represent the standard error of the mean of at least two independent experiments performed in duplicate. (B) Comparison of replication capacity over multiple rounds of infection for wild-type Ba-L versus mutant bearing the 4-amino-acid Gag matrix signature T122A/G123E/126del/127del. Error bars represent the standard errors of the means for technical replicates. Data are representative of two independent experiments.

A surrogate for fitness in our assay is single-round infectivity (measured in relative light units [RLU]) in the absence of drug, which is given a value of 100% for our reference subtype B virus. We measured the single-round infectivity (replication capacity [RC]) of clones bearing patient-derived *gag-pro* sequences from each time point. Interestingly, resistant clones had a lower RC than susceptible viruses (around 1.5-fold), regardless of whether they were isolated from VF1 or VF2 (Fig. 7). We also tested full-length replication-competent virus bearing the 4-amino-acid signature with the wild type over multiple rounds of replication and found a similar difference in RC (Fig. 7). The mixture of sensitive and resistant strains is consistent with incomplete drug adherence and therefore variable drug pressure, or alternatively with compartmentalization of virus sequences in anatomical areas with different drug levels.

## DISCUSSION

Based on NMR and X-ray crystallography studies, p17 comprises five major alpha helices connected primarily by short loops (35, 37). The C terminus of matrix is predicted to be disordered, which has hampered efforts to characterize the structural characteristics of this region. One study suggested that deletions at 125 and 126 would stabilize p17 (38), indicating that despite disorder, changes in the region might lead to significant changes in stability and therefore possibly altered effects of protease inhibition on cleavage.

In this study on CRF02_AG and subtype G clinical isolates from a Nigerian cohort, we demonstrated the role of p17 amino acid mutations occurring near the p17/p24 cleavage site in PI resistance. The double deletion of Ser and His at Gag positions 126 and 127, respectively, had a modest impact on *in vitro* phenotypic PI susceptibility. When this deletion occurred alongside T122A and G123E, we observed a 4- to 5-fold decrease in susceptibility to lopinavir. The four-mutation combination was also able to confer similar resistance to a subtype B virus, indicating that it may emerge across subtypes.

We aimed to understand the mechanism at play in the T122A/G123E/S126del/H127del phenotype. Western blotting of virus-containing supernatants from producer cells revealed significant differences in cleavage without drug at the p17/p24 cleavage site, with the resistant clone demonstrating more efficient cleavage. In the presence of drug, Gag cleavage at the MA/CA cleavage site (as obtained from p24/p41 ratios) was also more efficient in the resistant viral clone. Therefore, rescue of infectivity in the presence of drug *in vivo* might be explained by inherently more efficient or kinetically favorable cleavage.

G123E was reported to arise when viruses were propagated with investigational protease inhibitors KNI-272 and UIC-94003 (39). Gag G123E was found to potentially interact with protease by NMR (35), providing a potential mechanism for its effect. This was more recently corroborated by Samsudin and colleagues (40), using multiscale modeling and simulations to reveal how non-cleavage site mutations can directly interact with cleavage site residues to affect their local environment. Through the use of contact analysis between the MA/CA cleavage site residues and Gag position 123 in wild-type (WT) (G123) and mutant (E123) proteins, the residue at position 123 was shown to make contact primarily with the N-terminal portion of the cleavage site from the same Gag subunit. Both WT (glycine) and mutant (glutamate) residues showed a similar percentage of contact over the course of the simulations. When the CG simulations were “back-mapped” and transformed into atomic resolution, atomistic simulations of a single MA-CA-SP1 subunit showed that the glutamate (mutant) residue at position 123, but not the glycine (wild-type) residue, interacted primarily with the cleavage site Y132 and also contacted residues N131 and Q130. Given the change in the overall size and charge of the residue in the WT and mutants (from small and neutral to large and acidic), the G123E mutation alters the accessibility and electrostatic properties in the vicinity of the cleavage site and therefore was expected to directly interfere with proteolysis. Although our present study implicates G123E in reduced PI susceptibility, we show here that the combination of mutations that was observed in the patient was needed for maximal effect.

We next used NGS to explore the dynamics of emergence of Gag amino acid changes during ongoing viremia under PI treatment. We were able to detect both T122A and G123 at low abundance at baseline, prior to PI exposure. Importantly, PCR from plasma RNA using *gag*-*pro*-specific primers did not amplify any sequences with these changes at baseline, highlighting an important contribution of NGS to the study of drug resistance. Whole-genome reconstruction enabled us to infer phylogenetic trees and confirm findings that resistance-conferring mutations occurred at both time points in phylogenetically related sequences. All virus haplotypes were predicted to use CCR5 and therefore to be sensitive to the CCR5 antagonist maraviroc. Intriguingly, we found that resistant viruses had lower replication efficiency than the wild type in both single-round and multiround infections when there was no drug present. These experiments support a model where the composition of viral quasispecies under nonsuppressive ART depends on drug levels and inherent differences in replication dynamics conferred by relatively small numbers of amino acids.

Our study provides further information on the role of Gag in resistance to protease inhibitors. Given that failure of treatment with protease inhibitors arose with no major mutations in protease, the Gag protein itself could be a target for the development of future therapeutics. Presently, there are no FDA-approved antiretroviral drugs that target HIV-1 Gag. A number of studies have attempted to establish Gag as a target. The design and development of drugs that target Gag could be approached in four broad ways, as reviewed by Su and colleagues (41). The first approach would involve screening and targeting of druggable allosteric sites present in Gag. A second approach is the identification of novel Gag mutations and the use of models to preemptively design Gag inhibitors. The third approach is the use of synergistic drugs to target multiple sites by chemically joining different potential Gag inhibitors to function as dual or triple inhibitors. The fourth approach is to design inhibitors to disrupt the conformational transition of Gag during viral maturation (41).

The novel amino acid signatures that arose *in vivo* during treatment in the present study occurred in the matrix domain and at the non-cleavage site of Gag. The matrix (MA) domain of HIV-1 Gag plays critical roles in virus assembly by targeting the Gag precursor to the plasma membrane and directing the incorporation of the viral envelope (Env) glycoprotein into virions (42). A class of negatively charged lipids known as phosphoinositides, such as phosphatidylinositol-(4,5)-bisphosphate [PI(4,5)P_2_], play an important role in the association of HIV-1 Gag with the plasma membrane. To target the MA of Gag, small molecules could be synthesized either to bind to the PI(4,5)P_2_-binding cleft, thereby competing for an MA-P1(4,5)P_2_ association, or to target the hydrophobic groove in the globular core matrix, which would then dysregulate the myristyl switch mechanism and block the association of Gag with the cell membrane, thereby disrupting virus assembly and release (43). This mechanism could have been involved in the reduction in susceptibility by our four-amino-acid resistance signature in matrix. The use of a small-molecule approach was also adopted by Machara et al. and led to the identification of two arylquinazolines which inhibited HIV-1 capsid assembly by binding to the C-terminal domain of capsid and blocking viral replication (44). Additionally, inhibitors could be synthesized to destabilize Gag assembly, thus slowing the viral maturation process (41).

In future work, it would be interesting and important to know whether Gag mutations are capable of facilitating emergence of major protease mutations in prolonged culture conditions under suboptimal drug pressure. This could potentially explain why prevalence of major protease mutations increases over time during PI exposure in clinical studies (45). Next, one could perform population dynamics simulations to incorporate RC and susceptibility data in order to model the proportion of resistant and susceptible viruses over time and possibly therefore predict emergence of major mutations in the protease gene.

Our data are limited by the small sample size, the lack of availability of plasma drug level measurements, and the use of standard clonal approaches as opposed to single-genome sequencing and amplification. This meant that we were not able to assess the contribution of minority variant populations to susceptibility. Some experiments were done in duplicate rather than triplicate. Nonetheless, we hypothesize that the four-amino-acid HIV-1 Gag signature is a contributory factor in PI failure in this PLWH from Nigeria.

As we move toward next-generation sequencing, this work highlights the limitations of current genotyping methods to infer PI susceptibility and supports sequencing outside protease to broaden the evidence base for the clinical management of patients who experience VF on PIs without major protease mutations. The work may ultimately also help to identify define individuals with lower PI susceptibility before treatment with this class of drugs.

## MATERIALS AND METHODS

### Study participants.

We identified six individuals on second-line, protease inhibitor-based ART who experienced virological failure without major protease mutations from a PEPFAR-funded treatment cohort in Nigeria and who had samples collected at at least two time points (before second-line treatment initiation and following second-line virologic failure). Having previously reported that baseline phenotypic susceptibility was not associated with subsequent virologic “failure” (46) in this cohort, here we sought to explore changes over time in phenotypic susceptibility that could be associated with changes in HIV-1 Gag and protease genes.

### Next-generation sequencing.

Manual nucleic acid extraction was done using the QIAamp viral RNA minikit (Qiagen, Hilden, Germany) with a plasma input volume of 0.5 to 1.5 ml. The first strand of cDNA was synthesized using SuperScript IV reverse transcriptase (Invitrogen, Waltham, MA, USA), followed by NEBNext second-strand cDNA synthesis (E6111; New England Biolabs GmbH, Frankfurt, Germany). Sample libraries were prepared as per the SureSelect^XT^ automated target enrichment protocol (Agilent Technologies, Santa Clara, CA, USA) with in-house HIV baits. Whole-genome deep sequencing was performed using the Illumina MiSeq platform (Illumina, San Diego, CA, USA). Trimmed reads were then compared to a reference panel of 170 HIV subtypes/CRFs (circulating recombinant forms) from the Los Alamos database (https://www.hiv.lanl.gov), and the best match was used for reference mapping. Duplicate reads were removed from the BAM files, and a consensus sequence was generated using a 50% threshold. Mutations were included if they were present at a frequency greater than 2% within the read mixture at that position, with a minimum read depth of 100. An in-house custom script was used to identify single nucleotide polymorphisms (SNPs) at each position by BLAST analysis of individual HIV *pol* genes against the HXB2 reference genome.

### Haplotype reconstruction and phylogenetics.

Whole-genome haplotype reconstruction was performed using a newly developed maximum-likelihood method, HaROLD (haplotype assignment of virus NGS data using covariation of variant frequencies [47]). SNPs were assigned to each haplotype so that the frequency of a variant at any time point was represented by the sum of the frequencies of the haplotypes containing that variant. Time-dependent frequencies for longitudinal haplotypes were optimized by maximizing the log likelihood, which was calculated by summing over all possible assignments of variants to haplotypes. Haplotypes were then reconstructed based on posterior probabilities. The calculations were repeated with a range of possible haplotype numbers, and the optimal number of haplotypes was determined by the resulting value of the log likelihood. After construction of haplotypes, a refinement process remapped reads from BAM files to the constructed haplotypes. Haplotypes were also combined or divided according to Akaike information criterion (AIC) scores, in order to give the most accurate representation of viral populations. Phylogenetic trees of constructed haplotypes were constructed using RAxML-NG using the general time-reversible (GTR) model and 1,000 bootstraps.

### Coreceptor usage.

CCR5/CXCR4 usage was predicted using *env* sequences with the online tools Geno2Pheno (https://www.geno2pheno.org) and WebPSSM (https://indra.mullins.microbiol.washington.edu/webpssm/).

### Amplification of full-length Gag-protease genes.

We amplified from plasma taken before PI initiation and from a failure time point for each individual. NGS was used to obtain a consensus whole-genome sequence for each of the 12 samples. Full-length Gag-protease gene sequences were obtained from plasma by standard PCR; HIV-1 RNA was extracted from plasma samples using the QIAamp viral RNA extraction kit. Using previously described techniques (48, 49), the full-length Gag-protease sequence was amplified and cloned into a subtype B-based (p8.9NSX+) vector. Clonal sequencing of up to 10 plasmids was performed by standard Sanger sequencing. The variant that most closely represented the next-generation sequencing-derived consensus was taken forward for phenotypic testing. Sequences were manually analyzed using DNADynamo software (http://www.bluetractorsoftware.co.uk). Protease sequences were analyzed for PI resistance mutations using the Stanford Resistance Database (https://hivdb.stanford.edu). Phylogenetic analysis was performed using maximum-likelihood methods in MEGA v7.0 (50). Bootstrapping was performed as previously described (28).

### Site-directed mutagenesis.

Site-directed mutagenesis was carried out using the QuikChange kit (Stratagene) according to the manufacturer’s instructions. Mutagenesis was verified by Sanger sequencing.

### PI susceptibility and infectivity assays.

PI susceptibility and viral infectivity were determined using a previously described single assay. Briefly, 293T cells were cotransfected with a Gag-Pol protein expression vector (p8.9NSX) containing cloned patient-derived full-length Gag-protease sequences, pMDG (which expresses vesicular stomatitis virus envelope glycoprotein [VSV-G]), and pCSFLW (which expresses the firefly luciferase reporter gene with the HIV-1 packaging signal) as previously described. PI drug susceptibility testing was carried out as previously described (48). Transfected cells were seeded with serial dilutions of lopinavir, and harvested pseudovirions were used to infect fresh 293T cells. To determine strain infectivity, virus was produced in the absence of drug.

Infectivity was monitored by measuring luciferase activity 48 h after infection. Results derived from at least two independent experiments (each in duplicate) were analyzed. The IC_50_ was calculated using GraphPad Prism 5 (GraphPad Software Inc., La Jolla, CA, USA). Susceptibility was expressed as fold change in IC_50_ compared to that of the subtype B reference plasmid p8.9NSX. Replicative capacity of these viruses was assessed by comparing the luciferase activity of recombinant virus with that of the WT subtype B control virus in the absence of drug. Equal amounts of input plasmid DNA were used, and it has previously been shown that percentage infectivity correlates well with infectivity per nanogram of p24 in this system (48). Differences in PI susceptibility were compared with the paired *t* test.

### Multiround infectivity assay.

WT (R9-BaL) and mutant (R9-BaL with the 5 amino acid changes in MA) virus preparations were used to infect 1.5 × 10^6^ of SupT1-CCR5 suspension cells in 2 ml of medium per well and incubated at 37°C for 2 h, followed by low-speed centrifugation (800 × *g*) for 10 min. The supernatant was discarded, and the cell pellets were resuspended in RPMI medium and used to infect 4 × 10^6^ SupT1-CCR5 cells. Infectious virion supernatant was harvested on days 2, 4, 7, 9, and 11. The harvested virion supernatant was used to infect fresh TZM-bl cells to assay for infectivity, which was based on the Tat-dependent upregulation of long terminal repeat (LTR)-driven firefly luciferase expression upon HIV-1 infection of TZM-bl cells. Luciferase assay reagent was added, and the luminescence was measured using a GloMax 96 microplate luminometer (Promega).

The PI drugs used in this study were obtained from the AIDS Research and Reference Reagent Program, Division of AIDS, NIAID, NIH.

### Western blot analysis.

Using a previously described method (51), equal amounts of each of the viral clone plasmid were used to transfect 293T cells, in addition to a VSV-G plasmid and reporter genome-expressing plasmid. Each of the pseudovirions was produced in the absence and presence of a range of concentrations of LPV, added 16 h following transfection.

Forty-eight hours after transfection with the plasmid preparations, the culture supernatant was harvested and passed through a 0.45-μm-pore-size filter to remove cellular debris. The filtrate was centrifuged at 14,000 rpm for 90 min to pellet virions. The pelleted virions were lysed in Laemmli reducing buffer (1 M Tris-HCl [pH 6.8], SDS, 100% glycerol, β-mercaptoethanol, and bromophenol blue). Cell lysates were subjected to electrophoresis on SDS–4 to 12% bis-Tris protein gels (Thermo Fisher Scientific) under reducing conditions. This was followed by electroblotting onto polyvinylidene difluoride (PVDF) membranes. The HIV-1 Gag proteins were visualized by a transilluminator (Alpha Innotech) using anti-p24 Gag antibody.

### Ethics.

Informed consent was obtained from all participants, and ethics approval for virological testing was obtained from the Nigeria National Research Ethics Committee of Nigeria (NHREC/01/01/2007). Ethical approval was also obtained from the ethics board of University College London, United Kingdom.

### Data availability.

Sequences are available from GenBank under accession numbers MW125626 to MW125640.
