# Supplementary material for: In Vivo Emergence of a Novel Protease Inhibitor Resistance Signature in HIV-1 Matrix
Source: mBio. 2020 Nov 3;11(6):e02036-20. doi: 10.1128/mBio.02036-20 (PMC7642677; doi:10.1128/mBio.02036-20)
Supplement: FIG S1 [file mBio.02036-20-sf001.docx]

**Supplementary Figure 1:** **Phenotypic drug susceptibility to lopinavir of virus isolates at baseline (pre second line) and after failure of second line PI based ART.** Susceptibility is expressed as fold change in IC50 as compared to a subtype B reference in a single round assay. Error bars represent the standard error of the mean of at least two independent experiments performed in duplicate.

VF: viral failure
