# Supplementary material for: In Vivo Emergence of a Novel Protease Inhibitor Resistance Signature in HIV-1 Matrix
Source: mBio. 2020 Nov 3;11(6):e02036-20. doi: 10.1128/mBio.02036-20 (PMC7642677; doi:10.1128/mBio.02036-20)
Supplement: FIG S2 [file mBio.02036-20-sf002.docx]

**Supplementary Figure 2:** **Impact of protease mutation M46V on LPV susceptibility.** Mutations were made in both sensitive and resistant viruses. Data displayed are fold difference in IC50 values of LPV in comparison to that of the assay reference strain, p8.9NSX. Error bars represent the standard error of the mean of at least two independent experiments performed in duplicate.
