# Supplementary material for: In Vivo Emergence of a Novel Protease Inhibitor Resistance Signature in HIV-1 Matrix
Source: mBio. 2020 Nov 3;11(6):e02036-20. doi: 10.1128/mBio.02036-20 (PMC7642677; doi:10.1128/mBio.02036-20)
Supplement: FIG S4 [file mBio.02036-20-sf004.docx]

**Supplementary Figure 4: Gag 126del and 127del mutations occurring with T122A and G123E did not have a significant resistance effect on susceptibility to the protease inhibitor Darunavir** (A) The sequential introduction of mutations in the more susceptible clone (VF2 Clone 1) and (B), the reversion of the mutations in the resistant clone (VF2 Clone 2). The sequences of the viral clones showing the amino acid changes introduced using standard site directed mutagenesis are shown in red. Full-length Gag-protease amplified from plasma samples as well as respective mutants were VSV-g pseudotyped, encoding luciferase were produced by co-transfection in 293T cells. PI susceptibility of pseudovirions was determined using a single replication-cycle drug susceptibility assay as measured by luciferase activity. Data displayed are fold difference in IC50 values of LPV in comparison to that of the assay reference strain, p8.9NSX. Error bars represent the standard error of the mean of at least two independent experiments performed in duplicate.
