# Supplementary material for: In Vivo Emergence of a Novel Protease Inhibitor Resistance Signature in HIV-1 Matrix
Source: mBio. 2020 Nov 3;11(6):e02036-20. doi: 10.1128/mBio.02036-20 (PMC7642677; doi:10.1128/mBio.02036-20)
Supplement: TABLE S1 [file mBio.02036-20-st001.docx]

**Supplementary Table 1:** genotypic consensus level sequence data derived from next generation sequencing for six individual participants failing protease inhibitor based second line ART before (BL) and after virological failure (VF). Amino acids indicated represent differences from subtype B reference NL4.3. Amino acid positions in bold represent those previously associated with exposure to protease inhibitors.
