# Supplementary material for: In Vivo Emergence of a Novel Protease Inhibitor Resistance Signature in HIV-1 Matrix
Source: mBio. 2020 Nov 3;11(6):e02036-20. doi: 10.1128/mBio.02036-20 (PMC7642677; doi:10.1128/mBio.02036-20)
Supplement: TABLE S2 [file mBio.02036-20-st002.docx]

**Supplementary Table 2:** Representative raw IC50 data for sensitive (WT) and mutant (Mut2 with T122A, G123E, S125del, H126del) clones. Two replicates (1 and 2) are indicated with mean and SD.

| 1 | | 2 | Mean IC50 (nM) | SD |
| --- | --- | --- | --- | --- |
| sensitive | 4.79 | 4.36 | 4.575 | 0.30 |
| resistant | 17.104 | 17.63 | 17.367 | 0.37 |
