# Supplementary material for: In Vivo Emergence of a Novel Protease Inhibitor Resistance Signature in HIV-1 Matrix
Source: mBio. 2020 Nov 3;11(6):e02036-20. doi: 10.1128/mBio.02036-20 (PMC7642677; doi:10.1128/mBio.02036-20)
Supplement: FIG S5 [file mBio.02036-20-sf005.docx]

**Supplementary Figure 5: Role of single amino acid changes on LPV susceptibility.** (A) amino acid sequence alignment showing in red positions in a *gag-pro* sequence that confers ‘resistance’ mutated by site directed mutagenesis. (B) LPV fold change IC50 in a single round assay using luciferase as a read out. Error bars represent SE of mean of at least two independent experiments.
